# Surface enhanced Raman scattering of monolayer MX_2_ with metallic nano particles

**DOI:** 10.1038/srep30320

**Published:** 2016-07-26

**Authors:** Duan Zhang, Ye-Cun Wu, Mei Yang, Xiao Liu, Cormac Ó Coileáin, Mourad Abid, Mohamed Abid, Jing-Jing Wang, Igor Shvets, Hongjun Xu, Byong Sun Chun, Huajun Liu, Han-Chun Wu

**Affiliations:** 1 Elementary Educational College, Beijing key Laboratory for Nano-Photonics and Nano-Structure, Capital Normal University, Beijing 100048, P. R. China; 2 Key Laboratory of Cluster Science of Ministry of Education, School of Physics, Beijing Institute of Technology, Beijing 100081, P. R. China; 3 KSU-Aramco Center, King Saud University, Riyadh 11451, Saudi Arabia; 4 School of Physics and CRANN, Trinity College, University of Dublin, Dublin 2, Ireland; 5 Division of Industrial Metrology, Korea Research Institute of Standards and Science, Daejeon 305-340, South Korea; 6 Institute of Plasma Physics, Chinese Academy of Sciences, Hefei 230031, P. R. China

## Abstract

Monolayer transition metal dichalcogenides MX_2_ (M **=** Mo, W; X **=** S) exhibit remarkable electronic and optical properties, making them candidates for application within flexible nano-optoelectronics. The ability to achieve a high optical signal, while quantitatively monitoring strain in real-time is the key requirement for applications in flexible sensing and photonics devices. Surface-enhanced Raman scattering (SERS) allows us to achieve both simultaneously. However, the SERS depends crucially on the size and shape of the metallic nanoparticles (NPs), which have a large impact on its detection sensitivity. Here, we investigated the SERS of monolayer MX_2_, with particular attention paid to the effect of the distribution of the metallic NPs. We show that the SERS depends crucially on the distribution of the metallic NPs and also the phonon mode of the MX_2_. Moreover, strong coupling between MX_2_ and metallic NPs, through surface plasmon excitation, results in splitting of the 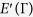 and 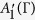 modes and an additional peak becomes apparent. For a WS_2_-Ag system the intensity of the additional peak increases exponentially with local strain, which opens another interesting window to quantitatively measure the local strain using SERS. Our experimental study may be useful for the application of monolayer MX_2_ in flexible nano-optoelectronics.

Two dimensional (2D) transition metal dichalcogenides (TMDs) have attracted much attention recently due to their outstanding electronic and optical attributes^1^,^2^,^3^,^4^,^5^,^6^,^7^,^8^,^9^,^10^. In contrast with graphene TMDs usually have energy bandgaps, ranging from 1 to 2 eV and typically display a transition from an indirect to direct-bandgap when the thickness is reduced to a monolayer^2^,^3^,^11^,^12^ For example, monolayer WS_2_ has a direct bandgap of 2.1 eV and its bulk state has an indirect bandgap of 1.3 eV^12^. The lack of inversion symmetry of TMDs together with strong spin–orbit coupling may lead to many as yet unforeseen applications^13^,^14^,^15^ Recent experimental studies suggest that monolayer WS_2_ and monolayer MoS_2_ have carrier mobilities of ∼140 cm^2^/(V·s) at low temperatures and a high on/off current ratio of 10^6^ 16,17,18. In particular, intense photoluminescence (PL) has been found in monolayer WS_2_^19^. These properties suggest that monolayer MX_2_ has the potential to be candidate within flexible 2D nano-optoelectronics. Mechanical strain is an important parameter in determining the performance of flexible devices^20^,^21^,^22^,^23^,^24^,^25^,^26^,^27^. It can strongly modify electronic structure and optical properties of 2D semiconductors. For example, a tensile strain exceeding 1% can cause a direct-to-indirect transition of the optical bandgap of monolayer MoS_2_^25^,^26^,^27^. Thus, to utilize monolayer MX_2_ within flexible 2D nano-optoelectronics, it is important to find a method to achieve a high optical signal, while quantitatively monitoring strain in real-time. Surface-enhanced Raman scattering (SERS) allows us to achieve both simultaneously. Raman spectroscopy is nondestructive and is widely used to measure the number of atomic layers, and the mechanical and thermal properties of graphene and various inorganic layered materials^28^. Moreover, the optical properties of monolayers such as graphene can be enhanced by surface resonances^29^,^30^,^31^,^32^,^33^, and similarly metals (e.g., Ag and Au) on the surface monolayer MX_2_ are known to produce enhancements through surface plasmon resonances^34^,^35^,^36^. However, the SERS depends crucially on the size and shape of metallic nanoparticles (NPs), which have a large impact on its detection sensitivity.

In this paper, we investigated the SERS of monolayer MX_2_, with particular attention paid to the effect of the distribution of the metallic NPs. We found that the SERS depends crucially on the distribution of the metallic NPs and also the phonon mode of the MX_2_. Strong coupling between the MX_2_ and metallic NPs, through surface plasmon excitation, results in splitting of the 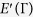 and 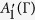 modes and an additional peak becomes apparent. For the WS_2_-Ag system, the intensity of the additional peak increases exponentially with local strain. Our experimental study may be useful for the application of monolayer MX_2_ in flexible nano-optoelectronics.

## Results

### Sample preparation and characterization

**Figure 1:**
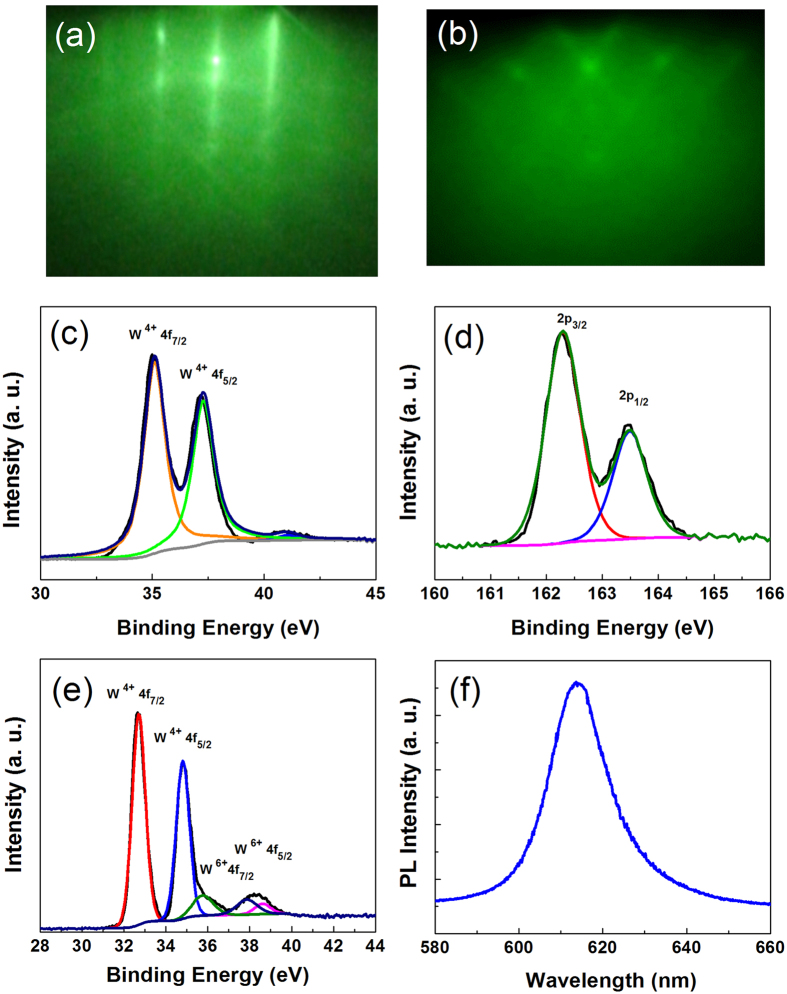
WS_2_ characterization. (**a**) RHEED patterns of a sapphire substrate after annealing with oxygen for 2 hours. (**b**) RHEED pattern of monolayer WO_3_ grown on a sapphire substrate. (**c**) X-ray photoemission spectroscopy compositional analysis of monolayer WO_3_ grown on a sapphire substrate. X-ray photoemission spectroscopy compositional analysis for the S (**d**) and W (**e**) peaks of monolayer WS_2_ grown on a sapphire substrate. (**f**) PL spectrum of monolayer WS_2_ grown on sapphire substrate.

Monolayer MX_2_ (M **=** Mo, W; X **=** S) can be obtained by the top-down methods, such as mechanical exfoliation^2^,^37^, and chemical exfoliation^38^,^39^,^40^,^41^, or by bottom up methods, such as transition metal sulfurization or metal oxide sulfurization^42^,^43^,^44^,^45^,^46^,^47^,^48^,^49^,^50^,^51^,^52^,^53^, and the decomposition of thiomolybdates^54^. In this work, to achieve large-scale continuous monolayer WS_2_ films, a monolayer WO_3_ film was first deposited on single crystal sapphire substrate using an e-beam heated WO_3_ source in a MBE system (DCA). Figure 1a shows a representative reflection high electron diffraction pattern (RHEED) of an α-Al_2_O_3_ (0001) substrate after annealing with an oxygen partial pressure of 1 × 10^−5^ Torr for 2 hours. The observed diffraction pattern of the [0110] crystallographic direction of the α-Al_2_O_3_ surface shows vertical lattice rods and sharp Kikuchi lines indicating a flat and well-ordered surface. The monolayer WO_3_ layer was deposited at a substrate temperature of 400 °C. After growth, the monolayer of WO_3_ was annealed at 650 °C with an oxygen partial pressure of 5 × 10^−6^ Torr for 30 minutes. Figure 1b is the RHEED pattern of a WO_3_ monolayer grown on the α-Al_2_O_3_ substrate after annealing. The sharp lines and diffraction spots originate from monoclinic WO_3_ (110) plane. One can see that WO_3_ can be epitaxially grown on a sapphire substrate which allows us to precisely control the thickness of WS_2_. The chemical composition of the WO_3_ was investigated by X-Ray photoelectron spectroscopy (XPS). Figure 1c shows that the W 4f_7/2_ and W 4f_5/2_ peaks are located at 35.1 eV and 37.2 eV indicating W^6+^ oxidation states. To form WS_2_, the sample was removed from the MBE chamber and sulfurized at 700 °C for 15 min in a furnace using 10% H_2_ in Argon gas as the carrier. Figure 1d,e show the XPS compositional analysis of monolayer WS_2_ grown on a sapphire substrate for the S and W peaks respectively. The peak positions for W^4+^ 4f_7/2_, W^4+^ 4f_7/2_, S 2p_3/2_, and S 2p_1/2_ are 32.7 eV, 34.7 eV, 162.3 eV, and 163.5 eV respectively. From Fig. 1d,e, we can also estimate that the atomic ratio of W: S is approximately 1: 2. PL spectra were used to further evaluate the thickness of the WS_2_ layer (Fig. 1f). A bandgap of ∼2 eV was observed indicating the grown WS_2_ was monolayer in nature^19^. To produce MoS_2_, a procedure similar to that for WS_2_ was used. After the growth of monolayer MoO_3_ by MBE, the sample was removed from the MBE chamber and sulfurized at 700 °C for 1 min to 2 min in a furnace. The chemical composition of the MoS_2_ layer was investigated by XPS (Figures S1a). The Mo 3d_5/2_, Mo 3d_3/2_, and S 2s peaks have been consistently energy shifted in order to position the peak in the C 1s region at a binding energy (BE) of 284.7 eV. The peak positions for the Mo 3d_5/2_, Mo 3d_3/2_, and S 2s are 229 eV, 232 eV, and 226 eV respectively, which are consistent with the values for bulk MoS_2_. From Figure S1a, we can also estimate that the atomic ratio of Mo: S is approximately 1: 2. Raman spectroscopy was used to further evaluate the quality of the MoS_2_ layer. Figure S1b shows high resolution transmission electron microscopy (HRTEM) of a MoS_2_ layer. The XPS and HRTEM results indicate the good quality and layered structure of the MoS_2_. As the area of the monolayer MX_2_ is determined by that of the initial MO_3_ monolayer, we developed a convenient technique to produce large-scale monolayer MX_2_. The overlaying Ag NPs of different sizes was achieved using E-beam evaporation. The size and the morphology of Ag NPs were characterized by SEM.

### SERS at room temperature

**Figure 2:**
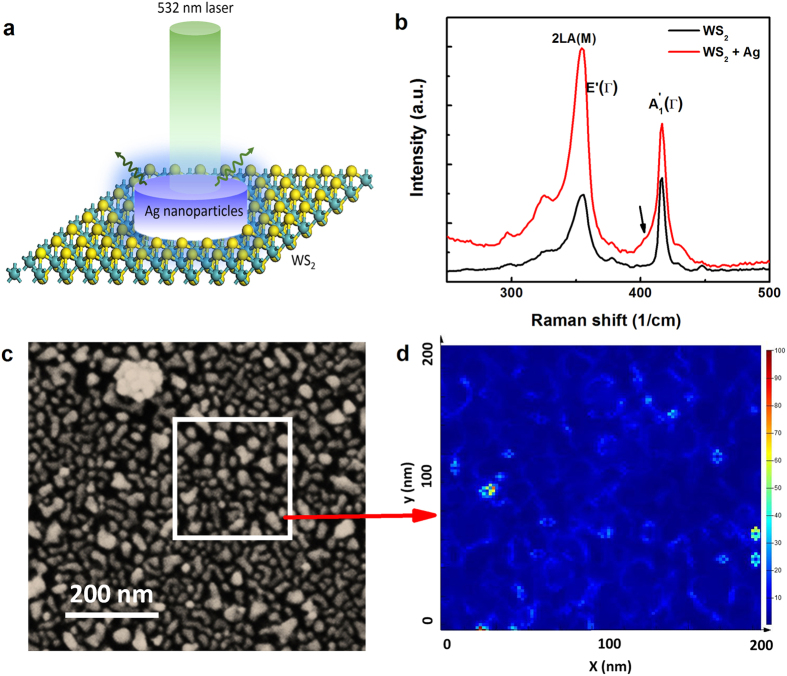
SERS of monolayer WS_2_ at room temperature. (**a**) Schematic drawing of the experimental setup for the SERS measurements of WS_2_. (**b**) Raman spectra of monolayer WS_2_ with (Red) and without (Black) 5 nm nominally thick Ag NPs on top. Arrow indicates the splitting of the 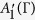 mode. (**c**) SEM image of 5 nm nominally thick Ag NPs on monolayer WS_2_. (**d**) Simulated electric field cartography at the surface of the area marked in Fig. 2c with an excitation wavelength of 532 nm.

Figure 2a shows a schematic of the experimental set-up. Raman measurements were performed using a Renishaw RM1000 spectrometer with an excitation wavelength of 532 nm. Figure 2b displays the Raman spectra of the monolayer WS_2_ without and with Ag NPs on top. Raman modes were assigned by fitting the spectra using a multi-peak Lorentzian approach as displayed in Figure S2. The Raman spectrum of monolayer WS_2_ displays an in-plane active mode 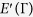 at 356 cm^−1^ and an out-of-plane mode 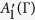
at 417 cm^−1^. The 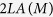 mode is located at 353 cm^−1^. Compared with bulk WS_2_^55^, the frequency of the out-of-plane mode 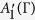 is red-shifted from 421 to 417 cm^−1^. The softening of the 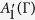 mode is due to the absence of Van der Waals interactions between the layers. By growing Ag NPs on the top of monolayer WS_2_, an increase in the intensity of the Raman active modes is observed, as shown in Fig. 2b. The intensity of the 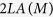
mode is enhanced by a factor of 3. Note, due to the non-uniform distribution of the Ag NPs, the enhancement factor has a variation of approximately 10%. Interestingly, for the out-of-plane 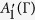 modes, additional peaks show up (marked with an arrow in Fig. 2b). In the vicinity of the metallic NPs, the electric field will be enhanced due to the collective oscillations of the conduction electrons inside the structure, thus the scattering process is enhanced by a factor of (E_local_/E_0_)^4^, where E_0_ is the strength of the incident E-field and E_local_ is the strength of the local electric field in the presence of the metallic NPs or at the plasmonic hotspots^34^,^35^,^36^. Moreover, the strength of the localized electric field depends on the size, shape and interactions between the isolated NPs. It is also reported that such an enhancement could be maximized if the excitation wavelength was tuned to be close to the surface plasmon resonance wavelength of the metal particles^56^,^57^,^58^. To understand how Ag NPs enhance the Raman signal of our monolayer WS_2_, the electric field amplitude cartography was simulated using a Finite Difference Time Domain (FDTD) approach. Figure S3a shows a schematic drawing of the structure used for the simulation. The position and shape of the silver NPs were extracted from SEM images by performing a simple filtering and thresholding processes (Figures S3b and S3c). All the simulations were performed using a mesh size of 0.2 nm in the x, y, and z-directions. Figure 2d shows the simulated electrical field distribution of the real surface with an excitation wavelength of 532 nm. At first glance, the electric field is localized at the Ag NP−WS_2_ boundary, indicating the enhanced Raman scattering at the Ag NP−WS_2_ boundary dominates the overall SERS signal which is consistent with other reports^36^. The amplitude of the normalized electric field intensity (|E_local_|^2^/|E_0_|^2^) varies from 3 up to 100, which corresponds to a local enhancement of the Raman scattering of 9 up to 10^4^. This heterogeneity is mainly due to the wide range of Ag NP sizes and shapes. Indeed, in the case of a Ag NP with an arbitrary shape, the polarizability of the NP is proportional to 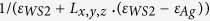 which is different from the resonance of an ideal sphere 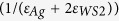, where 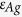 and 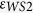 are the dielectric functions of the Ag NP and WS_2_ respectively. 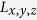 is the depolarization factor of the NPs in the x, y, and z-directions and 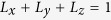 for an arbitrary shape. Moreover, when the distance between the nanostructures is decreased, strong electromagnetic coupling between the Ag NPs will occur which can further strengthen the electric field at the vicinity of interacting Ag NPs.

**Figure 3:**
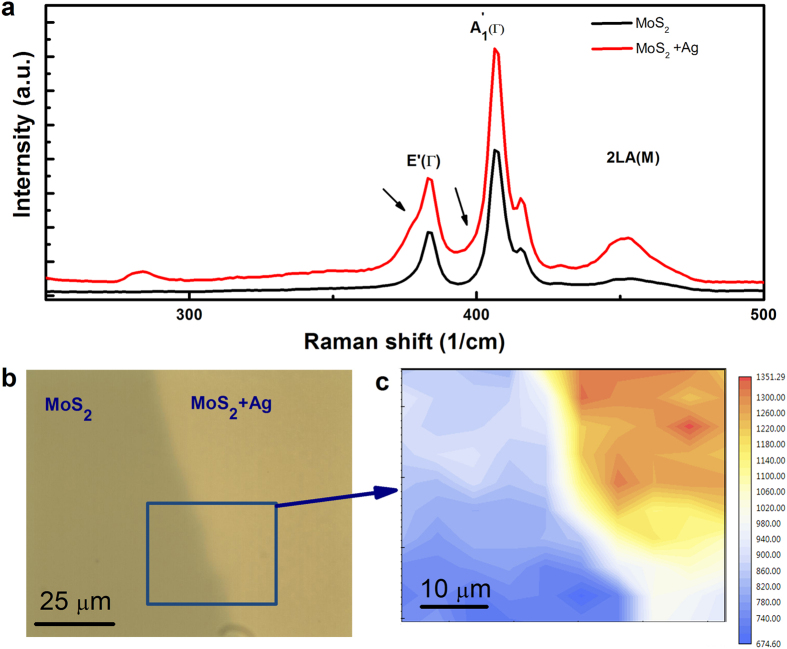
SERS of monolayer MoS_2_ at room temperature. (**a**) Raman spectra of monolayer MoS_2_ with (Red) and without (Black) 5 nm nominally thick Ag NPs on top. Arrows indicate the splitting of the 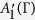 and 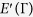 modes. (**b**) Optical image of MoS_2_ with and without Ag NPs on top. (**c**) 2D distribution of Raman intensity corresponding to the rectangle in Fig. 3b.

We observed similar effect in the Ag NP-MoS_2_ system. Figure 3a displays the Raman spectra of monolayer MoS_2_ without and with Ag NPs on top. By growing Ag NPs on the top of monolayer MoS_2_, an increase in the intensity of the Raman active modes is also observed. To clearly demonstrate the SERS effect, Fig. 3c shows the Raman intensity mapping of MoS_2_ with and without Ag NPs on top. Although a decrease in the intensity can be observed from top to bottom, due to absorbates or contaminants on the surface, the 2D Raman intensity mapping does correlate with the contrast seen in the rectangle in Fig. 3b. Thus, it gives another indication that the enhanced Raman signal is due to the SERS. Moreover, the intensity at the boundary of the bare MoS_2_ and MoS_2_/Ag is smaller than that of the MoS_2_/Ag but larger than that of bare MoS_2_, thus suggesting observation of the propagation of surface plasmons into the bare monolayer MoS_2_. Similar to WS_2_, the 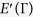, and 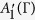 modes are split. Clear splitting of the 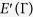
mode was not observed in Fig. 2b for WS_2_. The reason being that the 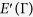 and *2LA(M)* modes for WS_2_ are very close. Moreover, the in-plane mode E_1g_ is activated, which is normally forbidden in the backscattering Raman process.

### Thickness dependent SERS

**Figure 4:**
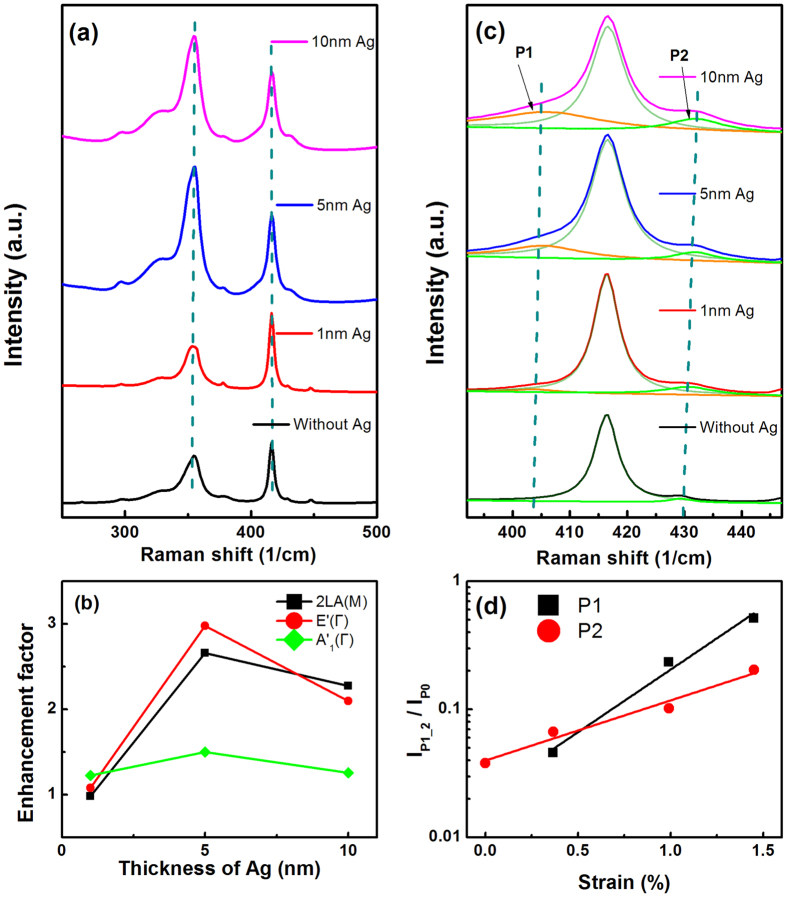
Thickness dependent SERS of monolayer WS2 and splitting of 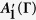 mode at room temperature. (**a**) Raman spectra of monolayer WS_2_ with different thickness Ag NPs on top. (**b**) Enhancement factors as a function of the Ag NP thickness. (**c**) Raman splitting of the 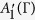 mode with different thickness Ag NPs on top of it. (**d**) I_P1_/I_P0_ and I_P2_/I_P0_ as a function of the Ag NP thickness induced strain.

To further investigate the impact of Ag NP distribution on the SERS signal, Fig. 4a shows Raman spectra of monolayer WS_2_ with different nominal thicknesses of Ag NPs on top. The Raman intensity does depend on the nominal thickness of the Ag NPs and the distribution of the Ag NPs. When the nominal thickness is increased from 1 nm to 10 nm, the morphology of Ag NPs is modified (Figure S4). For a thickness of 1 nm, well separated Ag NPs are present. When the thickness is increased to 5 nm, Ag NPs are separated but elongated and Ag NPs with multiple axes emerge. While for a thickness of 10 nm, it is more like a 10 nm thick non-continuous film on the surface. We define the enhancement factor as the ratio between the Raman intensity (frequency-integrated area under each peak) of a monolayer WS_2_ covered with Ag NPs and that without Ag NPs. Interestingly, SERS also depends on the phonon mode of the monolayer WS_2_. The enhancement factors of the 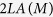, 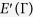, and 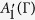 modes were 2.6, 3, and 1.5, respectively, for a 5 nm nominal thickness of Ag NPs (Fig. 4b). By increasing the nominal thickness of the Ag NPs from 1 to 5 nm, a clear enhancement of the Raman signal for the 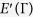 and 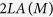 modes is observed, which is followed by a decrease in the enhancement factor when the thickness is increased to 10 nm. An explanation for the decrease in the enhancement factor for 10 nm nominally thick Ag NPs can be understood from the Ag morphology. The boundary density for the 10 nm non-continuous film is much less than that for 1 nm or 5 nm Ag NPs which decreases the overall SERS. The enhancement process is also accompanied by a red-shift of the Raman active modes, as shown in Fig. 4a. Interestingly, for out-of-plane 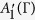 modes, the enhancement factor is less sensitive to the nominal thickness of the Ag NPs (Fig. 4b) and additional peaks show up at 405 and 432 cm^−1^ (Fig. 4c). It was shown by Wang *et al.* that due to strong surface plasmon excitation, the Raman signal at the boundary of metal-WS_2_ is dominated by local strain and the out-of-plane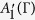 mode is split^36^. Similar effects can be expected in our Ag NP-WS_2_ system as the SERS signal is also dominated by the Raman scattering at the Ag NP-WS_2_ boundary. Moreover, the intensity of the split mode (P1 marked in Fig. 3c) increases linearly with the Ag NP nominal thickness (Figure S5). As the absolute intensity values depend on the laser power, we use I_P1_/I_P0_ where I_P1_ and I_P0_ are the intensities of modes P1 and P0 respectively. The intensity ratio has an error of 8% due to the Lorenztian fittings. To demonstrate it can be used to quantitatively measure the local strain, we plot the intensity of the split peak (P1) as a function of the local strain, where the local strain is quantitated by the relative shift of P0 (Table 1 in supporting information)^59^. In addition, from the error of the relative shift (±0.1 cm^−1^), the error for the calculated values of the local strain is approximately ± 0.3%. It is found that the intensity of the split peak increases exponentially with the local strain. Interestingly, the intensity of P2 also follows a similar strain dependence. In our Ag NP-WS_2_ system, strain is produced by the Ag NP deposition (Fig. 4d). The exponential dependence of the Raman intensity on the local strain opens another interesting window to quantitatively measure the local strain using SERS. It can also explain why the enhancement factor of the 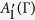 mode is less than that of the 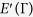 mode.

In conclusion, we investigated SERS for monolayer MX_2_, paying particular attention to the effect of the distribution of the metallic NPs. We show that the SERS depends crucially on the distribution of the Ag NPs and also the phonon modes of the MX_2_. Moreover, strong coupling between the MX_2_ and the Ag NPs through surface plasmon excitation results in splitting of the 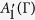 mode and additional peaks are manifested. The intensity of the additional peak increases exponentially with local strain, which opens another interesting window into quantitatively measuring the local strain through SERS.

## Methods

### Monolayer MX_2_ growth and characterization

To achieve large-scale continuous monolayer MX_2_ films, a monolayer MO_3_ film was first deposited on single crystal sapphire substrate using an e-beam heated MO_3_ source in a MBE system (DCA). Reflection high electron diffraction (RHEED) was used to monitor and establish growth mode. After growth, the monolayer of MO_3_ was annealed with an oxygen partial pressure of 5 × 10^−6^ Torr for 30 minutes. To form MX_2_, the sample was removed from the MBE chamber and sulfurized in a furnace using 10% H_2_ in Argon gas as the carrier. The quality of the sample was investigated by X-ray photoemission spectroscopy (XPS), Raman spectroscopy, high resolution transmission electron microscopy (HRTEM), and PL spectroscopy indicating the high quality of the monolayer MX_2_.

## Additional Information

**How to cite this article**: Zhang, D. *et al.* Surface enhanced Raman scattering of monolayer MX_2_ with metallic nano particles. *Sci. Rep.* **6**, 30320; doi: 10.1038/srep30320 (2016).

## Supplementary Material

Supplementary Information
