# Supplementary material for: Surface enhanced Raman scattering of monolayer MX2 with metallic nano particles
Source: Sci Rep. 2016 Jul 26;6:30320. doi: 10.1038/srep30320 (PMC4960528; doi:10.1038/srep30320)
Supplement: Supplementary Information [file srep30320-s1.pdf]

**Supporting Information**

# Surface enhanced Raman scattering of monolayer $MX_2$ with metallic nano particles

Duan Zhang[1,2,†], Ye-Cun Wu[2,†], Mei Yang[2], Xiao Liu[2], Cormac Ó Coileáin[2,3,4], Mourad Abid[3], Mohamed Abid[3], Jing-Jing Wang[4], Igor Shvets[4], Hongjun Xu[2,3], Byong Sun Chun[5], Huajun Liu[6], Han-Chun Wu[2,*]

[1]Elementary Educational College, Beijing key Laboratory for Nano-Photonics and Nano-Structure, Capital Normal University, Beijing 100048, P. R. China

[2]Key Laboratory of Cluster Science of Ministry of Education, School of Physics, Beijing Institute of Technology, Beijing 100081, P. R. China

[3]KSU-Aramco Center, King Saud University, Riyadh 11451, Saudi Arabia

[4]School of Physics and CRANN, Trinity College, University of Dublin, Dublin 2, Ireland

[5]Division of Industrial Metrology, Korea Research Institute of Standards and Science, Daejeon 305-340, South Korea

[6]Institute of Plasma Physics, Chinese Academy of Sciences, Hefei 230031, P. R. China

*Correspondence and requests for materials should be addressed to H.C.W. (wuhc@bit.edu.cn)

†These authors contributed equally to this work.

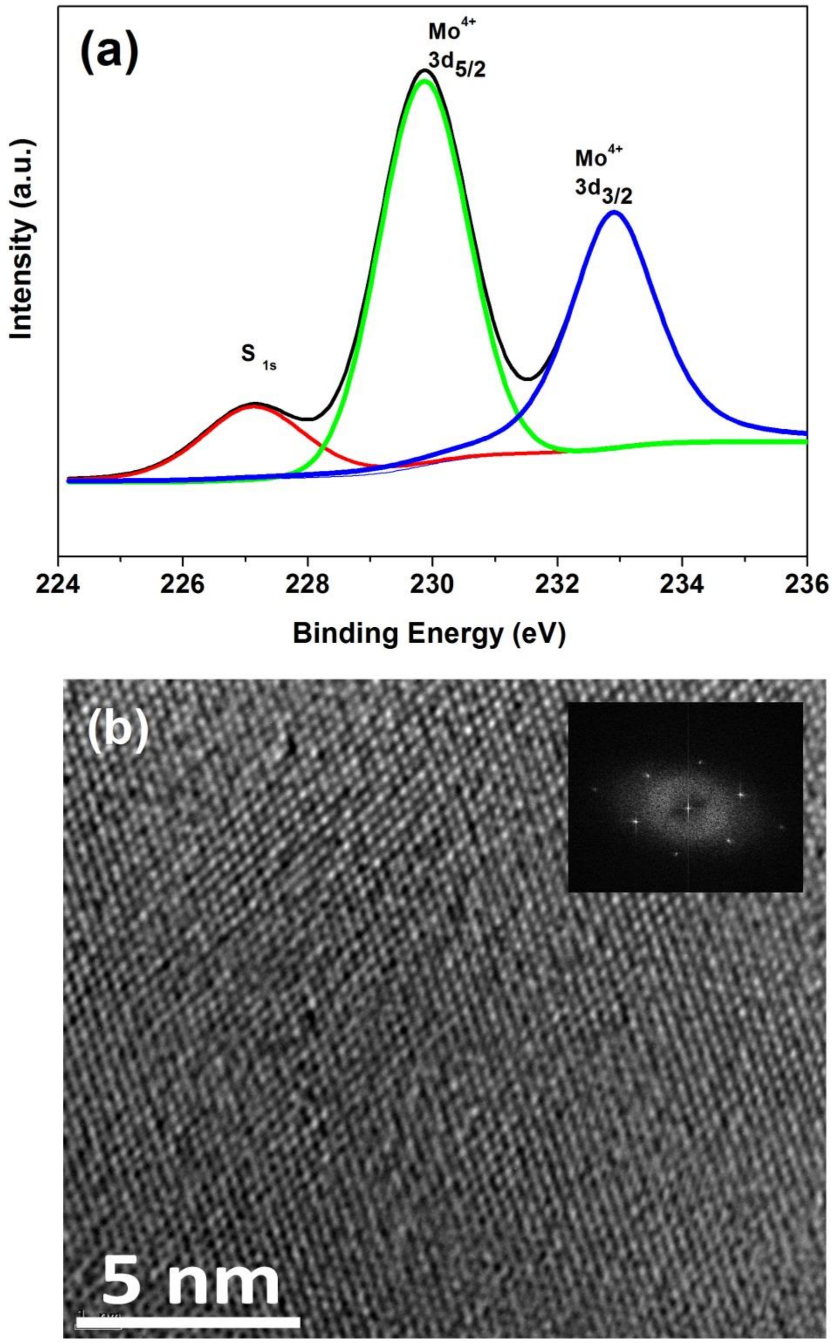


**Figure S1| $MoS_2$ characterization.** (a) X-ray photoemission spectroscopy compositional analysis of monolayer $MoS_2$ grown on a sapphire substrate. (b) HRTEM of monolayer $MoS_2$ grown on sapphire substrate to demonstrate the high quality of monolayer $MoS_2$. Insert: Electron diffraction pattern of the monolayer $MoS_2$ demonstrating the layer structure.

**Raman peak fitting:**

The Raman spectrum of monolayer $WS_2$ consists of a set of distinct peaks generated by different lattice vibrations and phonon dispersion. We performed the piecewise multi-peak Lorentzian fitting shown in **Figure S1** to obtain the individual peak characters.

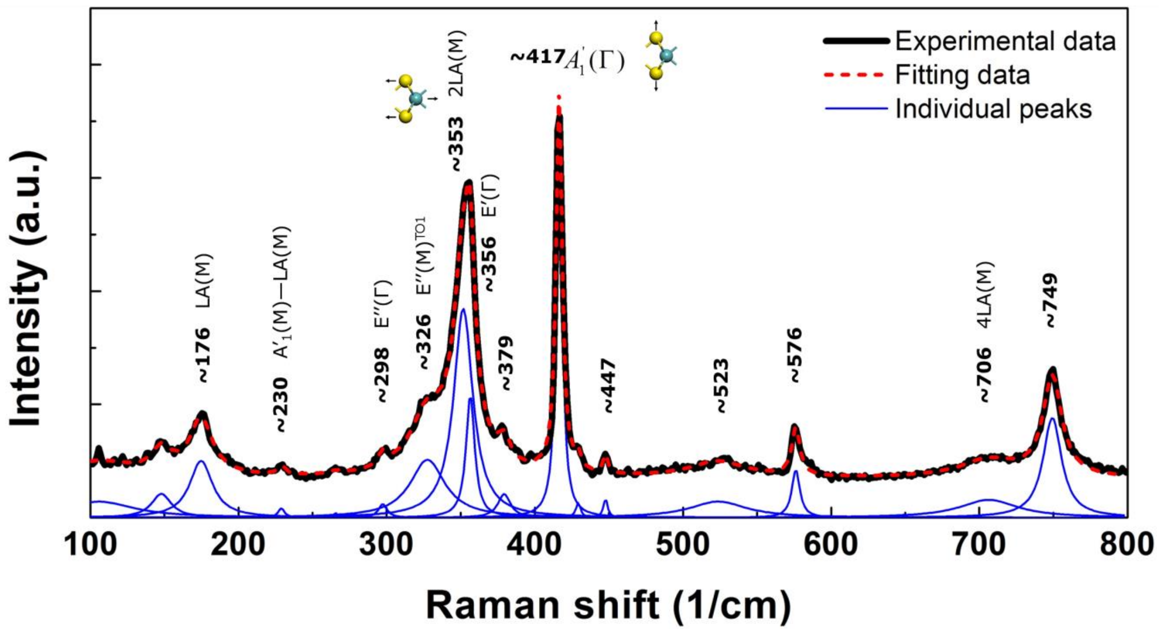


**Figure S2**. | Raman spectra of $WS_2$ without Ag using a 532 nm laser excitation at room temperature, including Lorentzian peak fits.

**FDTD simulation**

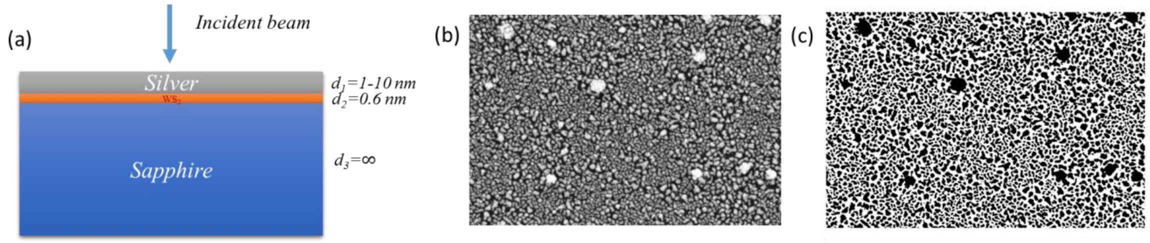


**Figure S3**. | (a) Schematic drawing of the structure used for FDTD simulation. (b) and (c) are the real SEM image and the image after the simple filtering and thresholding process respectively.

**Characterization of the Ag NPs**

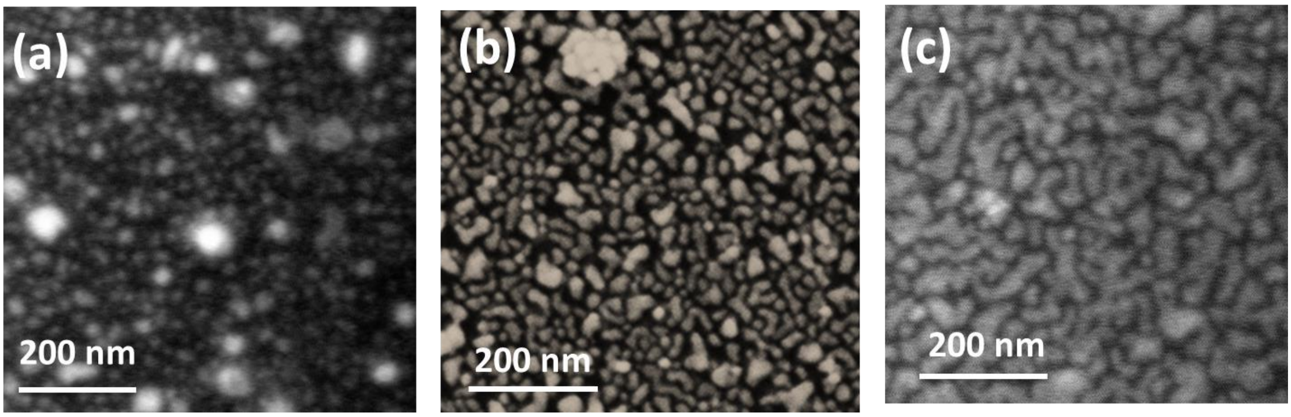


**Figure S4**. | SEM images of the Ag NPs' distribution for nominal thicknesses of 1 nm (a), 5 nm (b), and 10 nm (c).

| Ag NP thickness (nm) | Relative shift ($cm^{-1}$) | Local strain (%) |
|---|---|---|
| 0 | 0 | 0.00 |
| 1 | 0.11 | 0.36 |
| 5 | 0.30 | 1.00 |
| 10 | 0.43 | 1.44 |

**Table 1**: Correlation between Ag NP thickness and local strain.

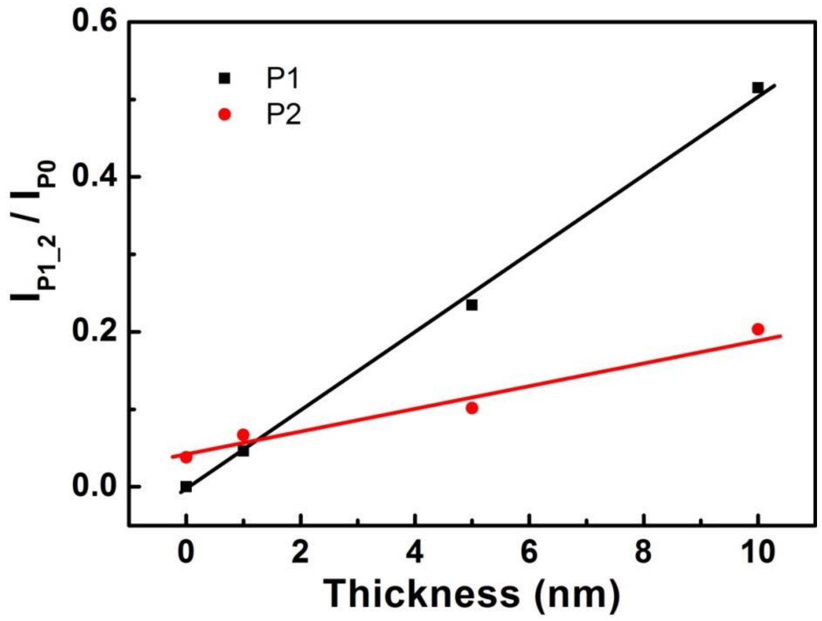


**Figure S5**. $I_{P1}/I_{P0}$ and $I_{P2}/I_{P0}$ as a function of the Ag NP thickness.
